# Trigeminal neuralgia due to Varicella-zoster virus reactivation

**DOI:** 10.1590/0037-8682-0118-2024

**Published:** 2024-07-29

**Authors:** Denise Jourdan Oliveira, Diogo Goulart Corrêa, Sérgio Ferreira Alves

**Affiliations:** 1 Universidade Federal Fluminense, Departamento de Radiologia, Niterói, RJ, Brasil.

**FIGURE 1:**
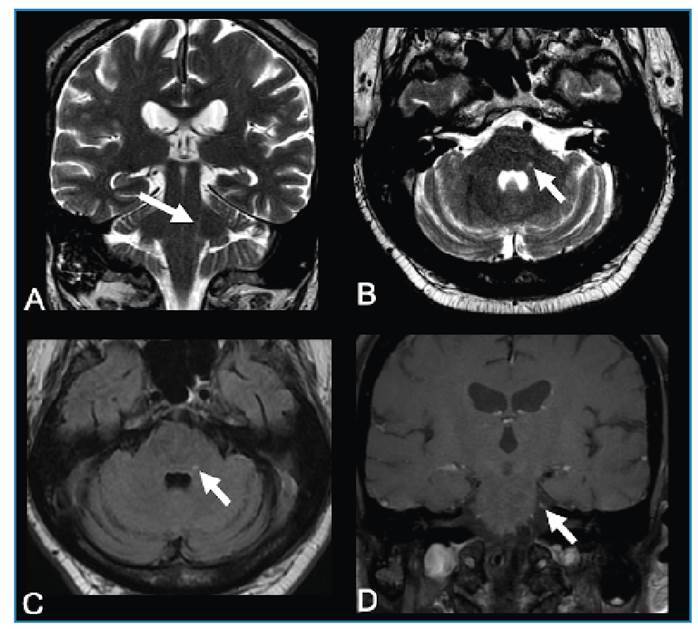
Trigeminal neuralgia due to varicella zoster virus reactivation. Brain MRI demonstrated a longitudinal lesion with hyperintense signal on T2-weighted imaging in the left spinal nucleus of the trigeminal nerve, in the coronal **(arrow in A)** and axial **(arrow in B)** planes and on an axial FLAIR **(arrow in C)**, associated with gadolinium enhancement in the cisternal segment of the left trigeminal nerve **(arrow in D)**. PCR of a cerebrospinal fluid sample revealed VZV positivity.

A 61-year-old man presented with left facial pain that worsened with digital touch and was associated with cutaneous vesicles in the left periorbital region for two weeks. Brain magnetic resonance imaging (MRI) revealed a longitudinal lesion with a hyperintense signal on T2-weighted imaging in the left posterolateral portion of the pons and medulla oblongata along the left spinal nucleus of the trigeminal nerve, associated with gadolinium enhancement in the cisternal segment (Figure 1). Cerebrospinal fluid (CSF) analysis revealed mild pleocytosis. Polymerase chain reaction revealed positivity for varicella zoster virus (VZV). Trigeminal postherpetic neuralgia was diagnosed, and the treatment comprised acyclovir, amitriptyline, and gabapentin, which reduced pain intensity and led to the progressive disappearance of the cutaneous lesions.

VZV is a herpesvirus whose primary infection causes varicella (chickenpox). Following primary infection, the virus becomes latent in the sensory neurons of the trigeminal ganglia and/or dorsal root ganglia along the neuraxis^1^. VZV reactivation is a common complication that occurs spontaneously or in response to several triggers, usually associated with aging and immunosuppression and, in some patients, may cause postherpetic neuralgia^2^. MRI may demonstrate gadolinium-enhancement of the trigeminal nerve or ganglion due to ganglionitis, and a hyperintense lesion on T2-weighted imaging along the trigeminal nuclei and intra-axial portion, including its entry zone, in patients with postherpetic neuralgia^3^. The spinal trigeminal nucleus extends from the inferior pons to the medulla and cervical spinal cord and receives sensory information regarding facial pain and temperature^4^. Thus, its involvement in VZV reactivation may explain the symptoms of postherpetic neuralgia.
